# Fast responses to stepping‐target displacements when walking

**DOI:** 10.1113/JP278986

**Published:** 2020-03-27

**Authors:** Yajie Zhang, Jeroen B. J. Smeets, Eli Brenner, Sabine Verschueren, Jacques Duysens

**Affiliations:** ^1^ Department of Rehabilitation Sciences FaBer KU Leuven Leuven Belgium; ^2^ Department of Human Movement Sciences Amsterdam Movement Sciences Vrije Universiteit Amsterdam Amsterdam The Netherlands; ^3^ Motor Control Laboratory Movement Control and Neuroplasticity Research Group FaBeR KU Leuven Leuven Belgium

**Keywords:** adjustment, gait, target jump, visual perturbation

## Abstract

**Key points:**

- Goal‐directed arm movements can be adjusted at short latency to target shifts.
- We tested whether similar adjustments are present during walking on a treadmill with shifting stepping targets.
- Participants responded at short latency with an adequate gain to small shifts of the stepping targets.
- Movements of the feet during walking are controlled in a similar way to goal‐directed arm movements if balance is not violated.

**Abstract:**

It is well‐known that goal‐directed hand movements can be adjusted to small changes in target location with a latency of about 100 ms. We tested whether people make similar fast adjustments when a target location for foot placement changes slightly as they walk over a flat surface. Participants walked at 3 km/h on a treadmill on which stepping stones were projected. The stones were 50 cm apart in the walking direction. Every 5–8 steps, a stepping stone was unexpectedly displaced by 2.5 cm in the medio‐lateral direction. The displacement took place during the first half of the swing phase. We found fast adjustments of the foot trajectory, with a latency of about 155 ms, initiated by changes in muscle activation 123 ms after the perturbation. The responses corrected for about 80% of the perturbation. We conclude that goal‐directed movements of the foot are controlled in a similar way to those of the hand, thus also giving very fast adjustments.

## Key points

- Goal‐directed arm movements can be adjusted at short latency to target shifts.
- We tested whether similar adjustments are present during walking on a treadmill with shifting stepping targets.
- Participants responded at short latency with an adequate gain to small shifts of the stepping targets.
- Movements of the feet during walking are controlled in a similar way to goal‐directed arm movements if balance is not violated.

## Introduction

The ability to adjust ongoing movements to new visual information has been studied extensively in goal‐directed reaching. When a target shifts to a new location, the hand can adjust its trajectory towards the new location within 100–160 ms (Oostwoud Wijdenes *et al*. 2013; Smeets *et al*. 2016; Zhang *et al*. 2018). It is not clear whether similar adjustments are to be expected for the cyclic movements of the feet when walking over a flat surface, because when walking the feet are carefully placed in the medio‐lateral direction to maintain balance (Donelan *et al*. 2004). However, the idea that reaching evolved from accurate limb positioning during locomotion, and the evidence that both behaviours are controlled using similar corticospinal circuits (Georgopoulos & Grillner, 1989; Yakovenko *et al*. 2011; Drew & Marigold, 2015; Yakovenko & Drew, 2015), suggest that similar circuitry might control all goal‐directed movements, resulting in similar fast adjustments.

Adjusting steps when walking is very common in daily life. Imagine walking along a muddy path, making sure to place the feet on the relatively dry parts. Where the foot is placed on each step is adapted to the terrain. If a disturbance happens during the leg swing, for instance because one placed one's foot on a slippery foothold on the previous step, foot misplacement or even a fall can occur. We know that it is possible to elicit foot placement adjustments. Such adjustments are not only found after mechanical perturbations (Hof & Duysens, 2013; Rankin *et al*. 2014; Afschrift *et al*. 2018), but also in response to unexpected obstacles (Moraes *et al*. 2004; Weerdesteyn *et al*. 2004; Potocanac *et al*. 2014 *b*) or to stepping stones being displaced (Bank *et al*. 2011; Peper *et al*. 2012; Young & Hollands, 2012; Hoogkamer *et al*. 2015; Mazaheri *et al*. 2015).

It is evident that one can adjust one's foot placement while walking, but it is unclear how fast such adjustments are. The latency of foot movement adjustments has been studied in step initiation from quiet stance to a target. In a study that examined attempts to reach a target when the target shifted about 20 cm laterally, Tseng *et al*. (2009) reported a response latency of about 250 ms. However, Reynolds & Day (2005) reported kinematic adjustments after only about 120 ms and electromyographic (EMG) adjustments after about 100 ms under quite similar circumstances, and slightly earlier (0–8 ms) when there was support. Kim & Brunt (2009) found a response latency between those of the other studies: 176 ms. However, these studies examined adjustments to a single step. Adjustments to visual perturbations during continuous walking have hardly been assessed. In a task in which participants were required to avoid obstacles while walking, Weerdesteyn and colleagues reported a response latency of 120 ms in the jerk (derivative of acceleration, Weerdesteyn *et al*. 2004), and on average 108 ms for changes in EMG (Weerdesteyn *et al*. 2007). For a sudden target shift during walking, Young & Hollands (2012) reported a latency of 200 ms for changes in foot trajectory.

We would classify kinematic adjustments of the foot with latencies well below 200 ms as fast adjustments (Smeets *et al*. 2016). Thus, while fast adjustments were reported for a reach‐like stepping task (Reynolds & Day, 2005), for walking only the study by Weerdesteyn *et al*. (2004) reports fast adjustments, and that study involved physical obstacles that could themselves perturb balance. Maybe quickly responding to perturbations was suppressed in other studies because such responses would perturb balance considerably since the perturbations, and therefore the required adjustments, were large. Adjusting foot positioning might be suppressed when such adjustments are not evidently essential, because not losing balance is prioritised (Barton *et al*. 2019). An example of such suppression has been reported for target shifts that were applied in a reach‐like stepping task: the magnitude of reported adjustments during step initiation were reduced if there was a balance threat (Reynolds & Day, 2005). Moreover, with large perturbations the step might be considered to be a different action rather than just an adjustment to the position (Smeets *et al*. 2016), and thus require ‘voluntary’ adjustments that might involve reprogramming the movement (reviewed for hand movements by Gaveau *et al*. 2014). To encourage fast responses of the foot, we therefore exposed walking participants to small target shifts that pose little threat to the participants’ balance.

Another related factor that might influence the presence of movement adjustments is the timing of the perturbation. Hand movement adjustments become stronger for perturbations closer to the end of the movement (Oostwoud Wijdenes *et al*. 2013; Smeets *et al*. 2016; Zhang *et al*. 2018), but this may not be so for foot adjustments because strong responses might lead to a loss of balance. In accordance with this possibility, the foot placement adjustments in response to late perturbations have been reported to be incomplete (Hoogkamer *et al*. 2015; Mazaheri *et al*. 2015) rather than stronger. We therefore considered the timing of the perturbation when analysing the data.

Thus, the question we have addressed is whether the leg muscle activation and the kinematics of the foot during walking can be adjusted with a latency that is clearly less than 200 ms in response to small changes in the position of stepping targets. We did this by projecting stepping targets on a treadmill moving at 3 km/h (0.83 m/s), occasionally shifting the target 2.5 cm to the left or right during the first half of the step, and analysing the kinematics and muscle activation of the leg. We also examined whether there are differences between medial and lateral adjustments. For step initiation, lateral corrections were larger than the medial ones and had a shorter latency: the earliest change occurred 114 ms after a lateral target shift but 121 ms after a medial target shift (Reynolds & Day, 2005). Finally, the question arises as to which muscles contribute to the adjustments, and in particular to what extent the response is bilateral, involving both the swing and stance leg. Based on earlier studies, one might expect that the activity of gluteus medius (GlM) is adjusted both on the swing side (Hof & Duysens, 2013; Rankin *et al*. 2014) and on the stance side (Hof & Duysens, 2013; Afschrift *et al*. 2018). Since these bilateral responses occur about synchronously, they seem to be part of a coordinated postural reaction rather than being an element of anticipatory postural adjustment.

## Methods

### Ethical approval

The study was approved by the Research Ethics Committee of KU Leuven (B322201732964), and was conducted in accordance with the standards set out in the *Declaration of Helsinki*, registered in the local clinical trial centre (clinical trial number at UZ Leuven: S60160). Each participant received information associated with experimental procedures, risks and potential benefits of participation before the enrolment, and then provided written informed consent.

### Participants

Twenty young adults (24.3 ± 3.6 years, 8 males) participated in this study. They all had normal or corrected‐to‐normal vision, and had no disease that is known to affect motor or sensory function. They had no problem with the task and were able to detect the small shifts of the stepping targets that occurred in an example video (see Supporting information Movie S1). All participants were self‐reported right‐leg dominant, as determined by asking them to imagine kicking a ball.

### Experimental set‐up

**Figure 1:**
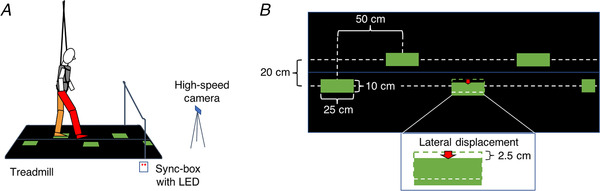
Set‐up *A*, side view of a participant who is standing on the left leg (orange). The right leg (red) is swinging to a green stepping‐target that shifts laterally. The belts of the treadmill are black and cover similarly sized force‐plates. For clarity, the projector and the motion capture cameras surrounding the measurement field are omitted from this picture. The high‐speed camera was used to determine the exact timing of the perturbation relative to the gait. *B*, top view of the treadmill with the same stimulus as in panel *A*. The red arrow indicates a 2.5‐cm lateral displacement of a stepping target for the right leg. [Color figure can be viewed at wileyonlinelibrary.com]

Participants walked at 3 km/h (0.83 m/s) on an instrumented treadmill (M‐Gait, Motekforce Link, The Netherlands). Two 3D force‐plates under the spilt‐belt treadmill measured the ground contact forces at 1000 Hz (Fig. 1*A*). A projector (Hitachi CP‐AW312WN LCD, Japan) projected stepping‐targets on the treadmill (green targets on black treadmill belts) from the right side of the participant at an angle of about 45 deg. We instructed the participant to step on the stepping‐targets, without any further instructions on how to walk. A safety harness prevented the participant from falling in the case of balance loss. Software (CueFors, Motekforce Link) triggered target displacements based on the gait pattern. We recorded the stimulus and the participant with a high‐speed video camera (Casio ER‐ZR 1000, Japan; sampling rate: 240 Hz) to determine the actual moment of target shift. The video was synchronised with the 3D motion caption system (Vicon, Oxford Metrics, UK, sampling rate: 200 Hz) by a box with LED lights, connected to an external trigger (sampling rate: 1000 Hz). We used the midpoint of a marker on the 2nd toe tip and a marker on the calcaneal to calculate the foot kinematics.

We recorded electromyographic (EMG) activity using a wireless system (Cometa Systems, Italy) at a sampling rate of 1000 Hz. We measured muscle activity from 8 muscles of each leg: gluteus medius (GlM), vastus lateralis (VL), vastus medialis (VM), biceps femoris (BF), semitendinosus (ST), tibialis anterior (TA), gastrocnemius lateralis (GaL), and gastrocnemius medialis (GaM). We will report EMGs of the swing leg (ipsilateral) and the stance leg (contralateral) separately. Electrodes were attached at positions following the recommendations of SENIAM (http://www.seniam.org/). Maximum voluntary contraction (MVC) was taken from the maximum of three trials of maximal isometric contraction for each muscle of each individual. To measure such contraction, a strap was fixed above the ankle in a sitting position for front upper leg muscles (to resist knee extension), and in a face‐down lying position with knee flexed (less than 90 deg) for back upper leg muscles (to resist knee flexion). The MVC of GlM was measured when the participant was lying on the side, and the strap was fixed above the ankle below the slightly flexed knee (to resist leg spreading). To test TA, the strap was fixed around the midfoot in a sitting position when the leg was supported (to resist ankle dorsiflexion and foot inversion). To test back lower leg muscles, the strap was fixed around the forefoot (to resist plantar flexion). Participants exerted forces against the strap for about 4 s while the examiner was encouraging them verbally. MVC was measured before or after the treadmill experiment.

The properties of stepping‐targets and their order of appearance were coded in Matlab (The MathWorks Inc, USA) and loaded to CueFors. The stepping targets were green rectangles (25 ×  10 cm). They were 50 cm apart in the direction of walking and 20 cm apart laterally (Fig. 1*B*). During some steps, the stepping target was displaced 2.5 cm, either medially or laterally. The shift was initiated when the participant's centre of pressure was 65 cm from the stepping target's position. This threshold of 65 cm corresponds to a moment early in the swing phase, and was considered to be a relatively easy level in previous studies (Hoogkamer *et al*. 2015, 2017; Mazaheri *et al*. 2015). As a result of delays in the equipment and variations in gait, the moment of perturbations varied within a range of about 200 ms with respect to the actual foot placement.

There were 10 walking episodes, each containing about 165 stepping‐targets (around 2 min of walking). There were 6 perturbations for each direction (medial or lateral) and for each leg (left or right) within a walking episode. These 24 perturbations were presented in a random order. The first 10 targets of each walking episode were always unperturbed. After that, a target with a perturbation occurred every 5–8 steps. As each participant performed 10 walking episodes, they had to deal with 240 perturbed targets (60 per combination of direction and leg) and around 1420 unperturbed targets (around 710 per leg).

### Procedure

Participants first walked normally on the treadmill for 1 min. After that, they were asked to step on a series of 120 unperturbed stepping‐targets when walking on the treadmill to practice placing their feet at indicated positions. They then performed the 10 walking episodes. They rested between episodes. They knew that the perturbations would be in the medio‐lateral direction, but did not know which step or which leg would be perturbed. They were instructed to step on the projected stepping targets as accurately as possible.

### Data analyses

#### Dependent variables

The variables describing the kinematics and centre of pressure (COP) that we report are signed: in the medio‐lateral direction, positive is in the same direction as the perturbation; in the anterior‐posterior direction, positive is in the walking direction. As a measure of adjustment accuracy, we defined the correction (%) as the medio‐lateral distance between the endpoint of a perturbed step and that of its reference (unperturbed control, see further below in References with no perturbation), divided by the size of the perturbation (2.5 cm). For instance, if a participant placed his or her foot 2.0 cm further in the perturbed direction after a perturbation, his or her correction was 80%. The lateral placement of the foot that was used to calculate this measure was its position at the moment of the next midstance (to ensure that the foot was flat on the treadmill).

**Figure 2:**
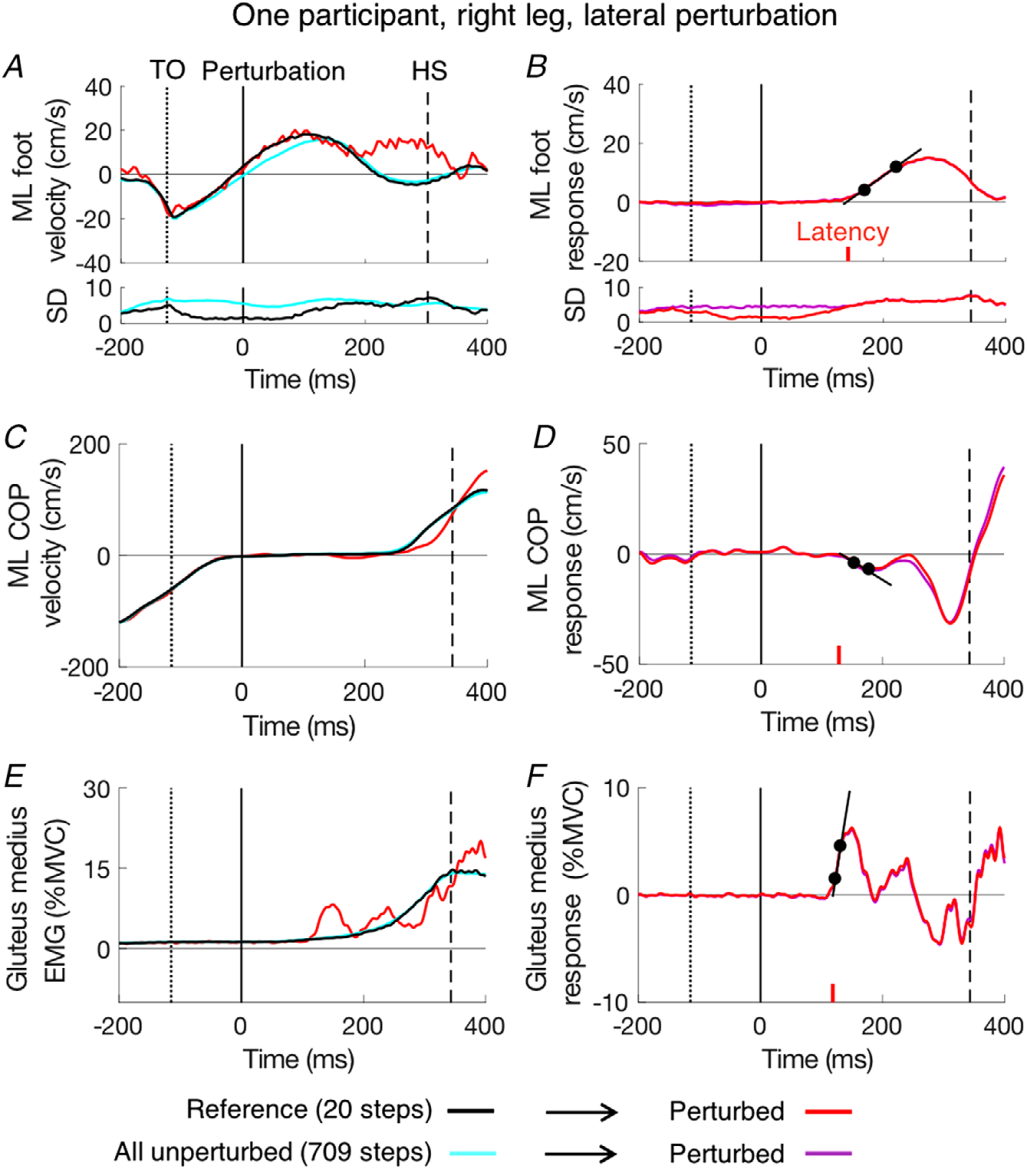
Example of a single participant's behaviour *A*, *C* and *E*, various actual values when the right leg was confronted with a lateral shift (red), in the matching steps during which it was not (black) and in all steps during which it was not (cyan). *B*, *D* and *F*, difference between values (responses) for steps with and without the lateral shift. This isolates the response (red) from which we can determine the latency (using the black lines; latency itself indicated by vertical red lines). *A* and *B*, lateral foot velocity. *C* and *D*, lateral velocity of change in centre of pressure. *E* and *F*, EMG of gluteus medius of the right (swing) leg. A single perturbed step is presented in panel *A* (red). All other red and purple traces show the means of the 60 perturbed steps. The curves in the lower parts of panels *A* and *B* represent the standard deviation (SD) of the mean across steps (709 steps for cyan, 20 for black and 60 for red and purple). The continuous vertical line indicates the moment at which the target shifts (time zero). The dotted and dashed vertical lines show the average moments of toe‐off and heel‐strike, respectively. The latencies in *B*, *D* and *F* are 142 ms, 128 ms and 115 ms, respectively, for this participant. Using all 709 unperturbed steps as the reference would have resulted in similar responses to using the selected reference at the overall level (purple *vs*. red, panel *D* and *F*), but it is not as accurate as using the selected reference at the single‐trial level (cyan *vs*. black, panel *A*). [Color figure can be viewed at wileyonlinelibrary.com]

The midpoint of toe and heel was used to describe the foot kinematics for the frontal and sagittal planes. Velocity was calculated using the central difference algorithm in the medio‐lateral and anterior‐posterior directions. Foot kinematic data were analysed unfiltered since they were smooth enough for latency calculation within each step. An example of one laterally perturbed step (red trace) and its unperturbed reference (black trace) is shown in Fig. 2*A*. We define the ‘response’ as the difference between perturbed steps and their references (Fig. 2*B*). The response isolates the effect of the target shift. The cyan and purple traces are only included to illustrate the benefit of our selection procedure and to show that this procedure does not introduce large biases. These traces show the reference (cyan) and responses (purple) when comparing perturbed movements with the overall mean movement on unperturbed trials as a reference, rather than with selected reference trials (as described in References with no perturbation). We obtain the response latency by drawing a line through the points at which the response reached 25% and 75% of the peak response, and taking its intersection with baseline (Veerman *et al*. 2008). We set the baseline as the averaged velocity from 50 ms before to 50 ms after the perturbation (which is very close to zero because this was the period used for matching the reference).

Bodyweight shifts in the medio‐lateral or anterior‐posterior direction were evaluated from shifts in the COP as measured by the force‐plates. Kinetic data were filtered using a zero‐lag 4th order low‐pass Butterworth filter with a cut‐off frequency of 20 Hz. Forces and moments were used to calculate the whole‐body COP. The COP data were noisier than the kinematics, so we could not determine the response in individual steps. The average COP velocity of perturbed and reference steps of the same participant is shown (Fig. 2*C*). The COP response (Fig. 2*D*) has two peaks, both in the direction opposite the foot response. We calculated the latency of the first peak (before 200 ms) in the same way as for the foot velocity.

EMG data was first band‐pass filtered (20–400 Hz, 4th order), then rectified, and then filtered again with a zero‐lag low‐pass filter (80 Hz, 4th order). Figure 2*E* shows the muscle activity in gluteus medius of the swing leg in perturbed and reference steps, and Fig. 2*F* shows the difference between the two: the ‘net’ muscle response to the perturbation. Muscle activity (%) was normalised to individual MVC level. The same extrapolation method was used to define the latency as for the other measures. Again, the latency was determined for the average response to a given perturbation. To make this possible, we had to decrease the low‐pass cut‐off frequency from 80 Hz to 30 Hz, because otherwise some of the data was too noisy. When comparing the response latency across kinematic, kinetic and EMG measures (Table 1), we used a measure of the latency for the lateral velocity of the foot that was also determined in this manner (per participant, after averaging the responses of the 60 steps with the same perturbation).

**Table 1:** Mean latencies with standard deviations (in ms) of the kinematic, kinetic and EMG responses with standard deviations across participants

	Lateral		Medial	
	Left	Right	Left	Right
Foot	156 ± 13 (20)	152 ± 10 (20)	164 ± 16 (20)	153 ± 13 (20)
COP	133 ± 21 (20)	127 ± 24 (20)	145 ± 22 (18)	129 ± 27 (20)
EMG				
i‐GlM	126 ± 9 (20)	120 ± 8 (20)		
c‐GlM	123 ± 11 (20)	121 ± 12 (20)	123 ± 17 (20)	123 ± 25 (20)
i‐ST	143 ± 22 (20)	136 ± 22 (20)	137 ± 19 (20)	130 ± 23 (20)

For the kinetic (COP) response in the Medial‐Left direction, two participants were excluded because they had no identifiable peak before 200 ms. Numbers of participants are given within brackets.

**Figure 3:**
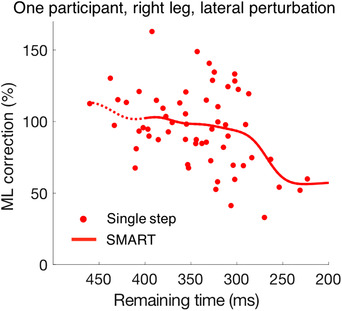
Data points for single steps and trace illustrating the trend The data (SMART method) are for the 60 perturbed steps that were analysed for Fig. 2 (one participant, right leg, lateral perturbation). The left part of the trace is dashed because in the further analysis (Fig. 8) we will only plot the remaining time from 400 to 200 ms as 95% of perturbed steps of all participants fell within this time window. [Color figure can be viewed at wileyonlinelibrary.com]

As the exact timing of the target shift within the step cycle varied from step to step, we checked how several kinematic variables depended on the time of the perturbation: the correction and the peak response velocity. Time was expressed as the ‘remaining time’, which for a perturbed step was the time from the moment of perturbation to heel‐strike (also termed ‘available response time’ in the literature). An example of the correction as a function of remaining time is shown in Fig. 3 for the 60 laterally perturbed steps of the participant whose data are shown in Fig. 2. The SMART method (van Leeuwen *et al*. 2019) was used to show the trend in the data points in a model‐free manner. The choice of the temporal resolution (standard deviation of the Gaussian kernel) had a negligible effect on the reconstructed time course; we chose σ = 18 ms. We analysed the responses as a function of the remaining time for perturbations within the first half of the leg‐swing (from 400 to 200 ms) as 95% of perturbed steps fell within this time window. We combined the data of the left and right leg since responses were basically similar (except for a small difference in latency; see further) and compared the differences between responses to medial and lateral perturbations using the SMART method. EMG data are sometimes shown for the stance leg as well as the swing leg.

Latencies were determined from the mean response (Fig. 2*B*, *D* and *F*) for each leg and perturbation direction of each participant, and subsequently averaged across participants.

#### References with no perturbation

We used the large set of unperturbed steps to search for the medio‐lateral foot velocity profile that matched each trace of the perturbed steps as closely as possible near the time of the perturbation. The selection was done from a pool of all the about 710 unperturbed steps for the swing leg of that participant. For each perturbation, the 20 steps that were most similar in their lateral velocity profile to the perturbed step between 50 ms before and 50 ms after the perturbation were selected. Similarity was defined as the smallest Fréchet distance (using the code by Ursell, 2013). We used the mean of the set of 20 selected unperturbed steps as the reference for the corresponding perturbed step for all our analyses, including the analysis of the COP and the EMG. An example of the effect of using a selection compared to using all unperturbed steps is shown in Fig. 2. The selection yielded a much better reference at the level of the individual step (compare black and cyan traces in Fig. 2*A*) without introducing evident biases in the average data (red and purple traces overlapped in Fig. 2*B*).

### Statistics

Data are reported as means ± standard deviation across participants (*n* = 20). Two participants were excluded for the latency of kinetic (COP) response in the medial‐left direction because they had no identifiable peak before 200 ms. Results for each reported muscle include data from 20 participants except VL and VM, which were from 19 participants (a few channels did not record properly during some measurements). A 2 ×  2 ANOVA was used to evaluate whether the amount of correction depended on the kind of perturbation (medial/lateral) or on the leg (left/right). *P* < 0.05 was considered as significant.

## Results

All adults in this study were able to make appropriate medio‐lateral adjustments. A small (2.5 cm) shift of a target induced a remarkably strong response of the foot while walking. The swing durations were very consistent across participants: on average the swing duration from toe‐off to heel‐strike was 397 ± 14 ms for unperturbed steps, and 403 ± 15 ms for perturbed steps. The precision of the stepping movements, expressed as lateral position of the foot at mid‐stance, was 1.0 ± 0.2 cm for the unperturbed steps. Perturbations were imposed with a considerable trial‐to‐trial variability within participants (see section Data analyses), but at a consistent average time across participants: 113 ± 8 ms after toe‐off. The responses to the target‐shifts did not induce any visible balance problems.

**Figure 4:**
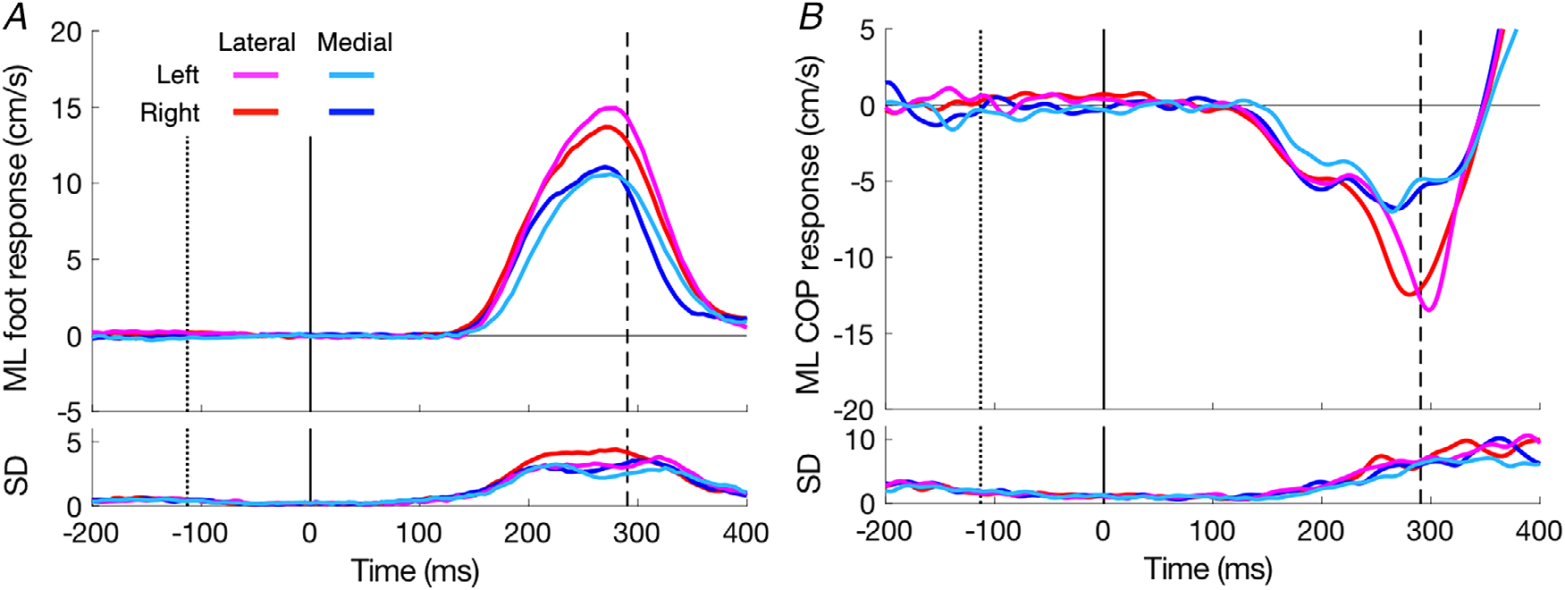
Medio‐lateral responses to target shifts as a function of the time after the perturbation *A*, foot responses. *B*, centre of pressure responses. Positive responses correspond to a change in velocity in the same direction as the perturbation. Curves in the upper part are the average responses; the ones in the lower part show the SD across the 20 participants. Vertical lines show the average onset (toe‐off, dotted) and offset (heel‐strike, dashed) of the leg‐swing, and the moment of perturbation (continuous line at time zero). [Color figure can be viewed at wileyonlinelibrary.com]

As expected, the foot (swing leg) responded in the same direction as the perturbations (Fig. 4*A*). This response is accompanied by a response of the centre of pressure (COP, stance leg) in the opposite direction (Fig. 4*B*). The magnitudes of both responses are larger for lateral perturbations (red and magenta traces) than for medial perturbations (blue and light blue traces). Consequently, the mean lateral correction was larger for the lateral (76 ± 18%) than for the medial (58 ± 12%) perturbation (*F*_(1,76)_ = 23.899, *P* < 0.001). The two legs showed no difference in correction (*F*_(1,76)_ = 0.008, *P* = 0.931). Table 1 shows that the latencies differ between the legs. Overall, the right leg appeared to respond slightly faster (8 ± 15 ms) than the left leg. The COP responded about 23 ms earlier than the foot kinematics, and the c‐GlM responded 11 ms earlier than the COP. We did not perform ANOVA (perturbation side, medial/lateral; leg side, left/right; variable type, kinematics/kinetics/EMG) to test the difference between medial and lateral response latencies: this is explained in the Discussion section.

**Figure 5:**
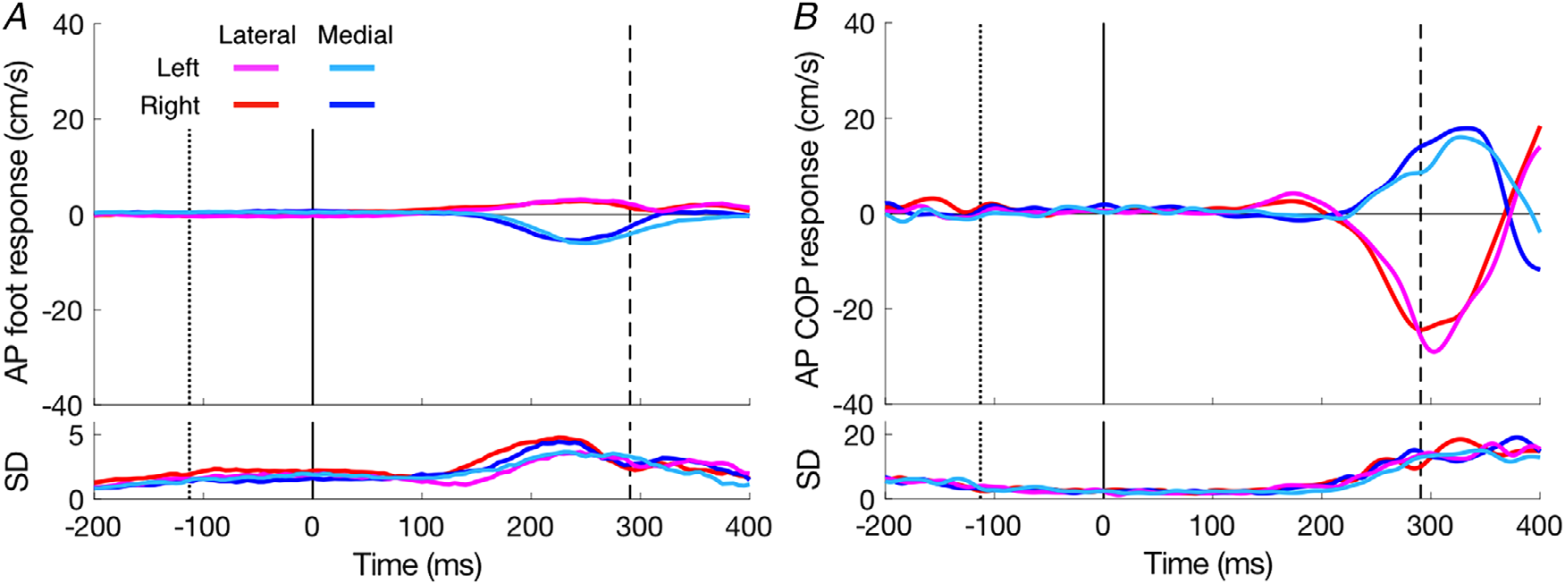
Average responses of the 20 participants in the anterior‐posterior direction, perpendicular to the medio‐lateral target shifts *A*, foot responses. *B*, centre of pressure responses. Positive responses are in the anterior direction. Other details as in Fig. 4. [Color figure can be viewed at wileyonlinelibrary.com]

We also took a look at the foot and COP responses in the anterior‐posterior direction (Fig. 5*A* and *B*, respectively). Though this was not the perturbation direction, some clear adjustments were observed, especially in the COP. The foot responses for lateral perturbations were too small to reliably determine a latency. In contrast, the COP responded strongly in the anterior‐posterior direction with a latency of about 225 ms (Fig. 5*B*). This strong response was opposite to that of the swing foot, as it was for COP responses in the medio‐lateral direction. For lateral perturbations there also appeared to be a small earlier (150–200 ms) COP response in the same direction as the foot.

**Figure 6:**
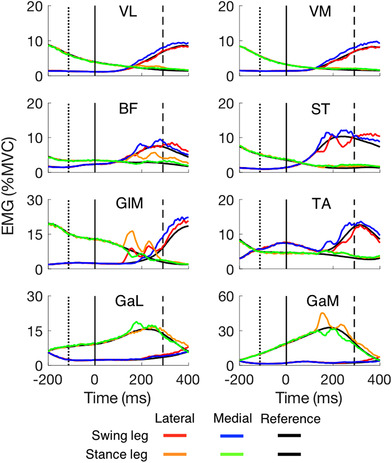
The EMG of all 8 muscles for steps with medial and lateral target shifts Responses are averages across 19 participants for VL and VM, and across 20 participants for the other 6 muscles. EMG magnitude is shown for both the swing leg and stance leg as a function of time after the target shift. The EMG in the corresponding reference steps is indicated by the two black traces, sometimes hidden behind the coloured traces. VL, vastus lateralis; VM, vastus medialis; BF, biceps femoris; ST, semitendinosus; GlM, gluteus medius; TA, tibialis anterior; GaL, gastrocnemius lateralis; GaM, gastrocnemius medialis. [Color figure can be viewed at wileyonlinelibrary.com]

**Figure 7:**
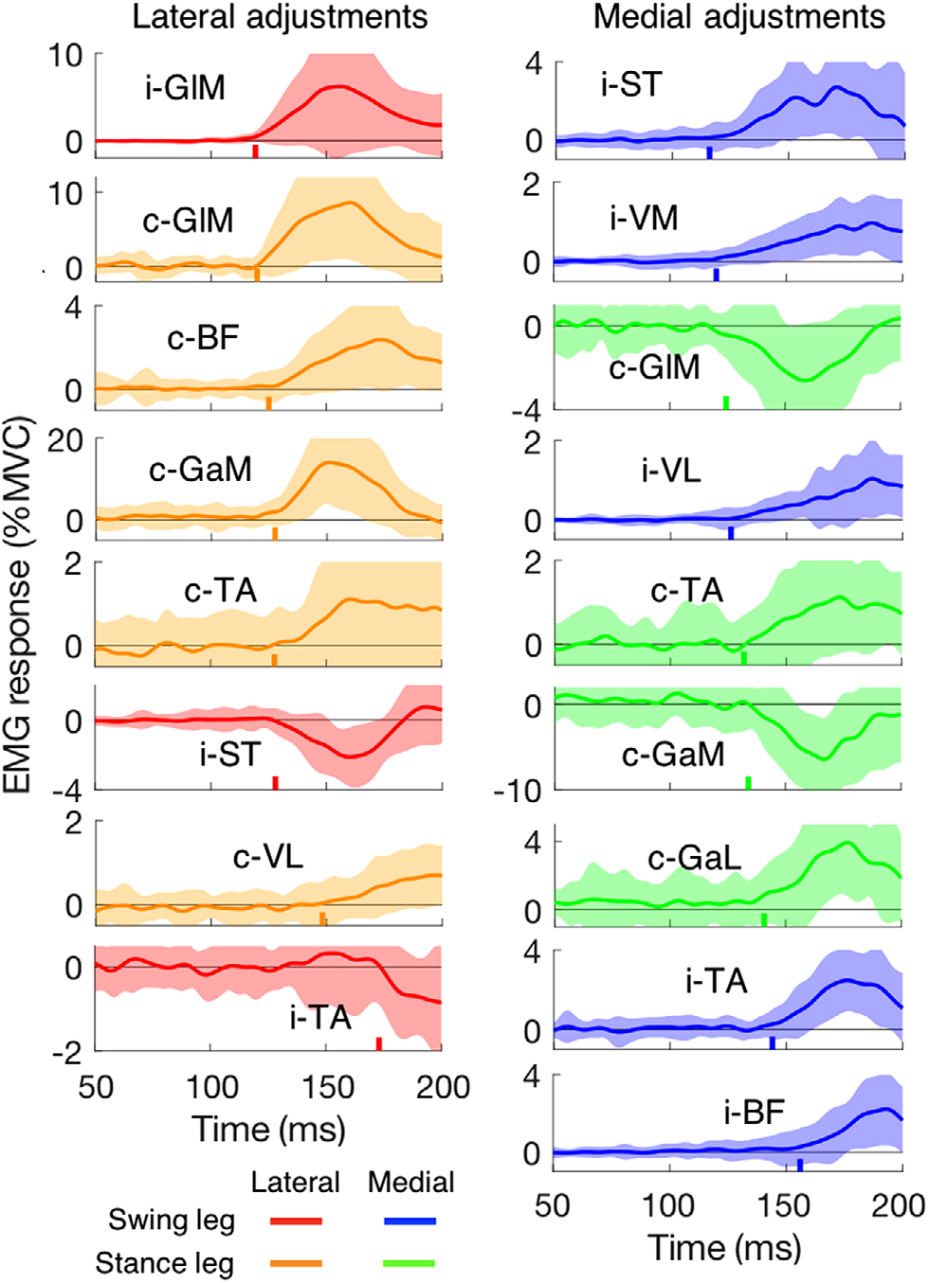
Main muscle activity changes in response to target shifts as a function of the time after the perturbation Curves are averages; shaded areas represent the SD across the 20 participants (19 participants for i‐VM and i‐VL). Muscles are ordered by the latency of their response (indicated by a coloured vertical line on the time axis). Left column: lateral target shift. Right column: medial target shift. The ‘i’ and ‘c’ in front of the muscle names indicate ipsilateral (the swing leg) and contralateral (the stance leg), respectively. Muscle abbreviations are detailed in Fig. 6. Note that the latencies indicated are based on the average response rather than on individual responses as in Table 1. [Color figure can be viewed at wileyonlinelibrary.com]

**Figure 8:**
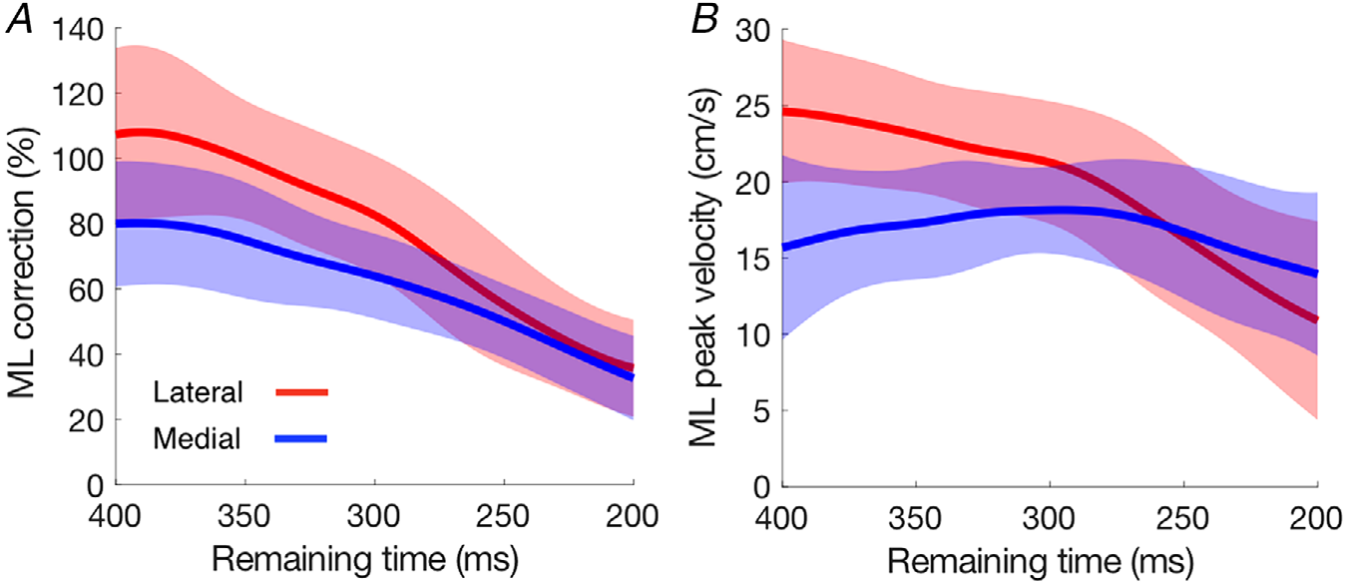
How the medio‐lateral response to a medial or lateral target shift depends on the remaining time until heel‐strike *A*, correction. *B*, peak velocity. Shaded areas represent the SD across the 20 participants. [Color figure can be viewed at wileyonlinelibrary.com]

Given the similarity between the two legs in the kinematic and kinetic responses (Figs 5, 6, 7, 8) and the generally quite small latency differences between the legs seen in Table 1, we will present EMG data pooled over the two legs (Fig. 6). The EMG analysis aims to evaluate the temporal order of the responses of various muscles. We had no reason to expect the two legs to use different strategies. The background EMG activity of all 8 muscles varies as a function of time, both for the swing leg and for the stance leg. At the time of the perturbation, all muscles were more active in the stance leg than in the swing leg except for the tibialis anterior (TA). This pattern reverses during the swing, so that at the time of heel‐strike, all muscles were more active in the swing leg than in the stance leg except for both heads of the gastrocnemius (medial, GaM; lateral, GaL).

Most muscles changed their activity in response to the target shift. For the stance leg (contralateral to the shift), the activity of the biceps femoris (BF), gluteus medius (GlM), and GaM increased after a lateral target shift (orange traces). The activity of GlM and GaM of the same leg decreased after a medial target shift (green traces), whereas that of the GaL increased. For the swing leg (ipsilateral to the shift), the activity of GlM increased after a lateral target shift (red trace), whereas that of semitendinosus (ST) and tibialis anterior (TA) decreased. The activity of ST, BF and TA of the same leg increased in response to a medial target shift (blue traces). The activity of vastus lateralis (VL) and vastus medialis (VM) did not change much in response to a target shift.

Response latencies of some of the earliest responding muscles were calculated for each participant, and averaged. The gluteus medius responded at a similar short latency in both the stance leg and the swing leg, on average 123 ms after the target shift (Table 1). For all muscles for which the trace in Fig. 6 differs substantially from the reference we also plotted the differences to isolate the responses to the shifts. We did so independently for both lateral and medial perturbations (Fig. 7). It is clear that GlM and GaM show the largest changes in muscle activity: about 10% of their maximal activation. For lateral perturbations, the earliest response could be found in the GlM of both the ipsi‐ and contra‐lateral leg. The earliest muscle response to medial perturbations is visible in ST of the swing leg. This muscle showed suppressed activity after lateral perturbations. Ipsilateral VM and VL responded with small changes but the changes occurred quite early (around 123 ms). Unlike the increase in activity in response to lateral perturbations, the activity of the contralateral GlM was suppressed in response to medial perturbations. For both directions of perturbation, the activity of TA of the stance leg increased at about the same latency (around 130 ms) as the gastrocnemii.

As we reported in the beginning of the Results section, the magnitude of the correction was smaller for target shifts in the medial direction than in the lateral direction. The question arises whether this difference is based on a difference in magnitude or in vigour. If the magnitude is limited, one might expect the difference to disappear when there is not enough time to make a large correction anyway. If the vigour is the limiting factor, one might expect the difference to be most prominent when the corrections have to be made quickly because there is limited remaining time. As expected, the magnitude of the correction decreased as the remaining time decreased. The difference between medial and lateral perturbations also decreased. It was absent when there was less than 250 ms left to respond (Fig. 8*A*). We see a similar pattern for the peak in the response velocity (Fig. 8*B*). Thus, the difference between responses to medial and lateral perturbations appears to be related to the magnitude rather than the vigour of the response.

## Discussion

In this study, we found clear fast adjustments of foot movement in response to target shifts. The foot adjustments had a latency of about 155 ms. We found responses in centre of pressure (COP) that had a latency of about 133 ms and changes in muscle activity that had a latency of about 123 ms. The contralateral (with respect to the foot for which placement had to be adjusted) gluteus medius (GlM), gastrocnemius medialis (GaM) and tibialis anterior (TA) responded quickly to both kinds of medio‐lateral perturbations. The ipsilateral GlM and contralateral biceps femoris (BF) responded quickly to lateral perturbations, and the ipsilateral semitendinosus (ST) responded quickly to medial perturbations. The muscle responses correcting for lateral target shifts were larger than those for medial shifts, in line with the larger foot placement corrections made in the lateral direction.

### Behavioural responses

The latency of medio‐lateral foot adjustment in our study (155 ms) was short, but the response of the COP had an even shorter latency (on average 133 ms). Is this difference because the COP shifts in anticipation of the kinematic correction? We argue here that this is not the case and will provide supporting evidence based on the EMG data in the Muscle responses section. When trying to compare our values with those of other studies, we find a wide variety of values for the fastest response, ranging from 120 ms (Weerdesteyn *et al*. 2004) to 200 ms (Young & Hollands, 2012). These differences can largely be explained by differences in methodology, such as the use of different methods to determine latency. An important choice when determining the latency of an adjustment is what signal to use: the higher the derivative, the shorter the resulting latency (Oostwoud Wijdenes *et al*. 2014; Brenner & Smeets, 2019). As the timing of changes in the COP has been shown to correspond to that of the acceleration of the centre of mass (Reimann *et al*. 2018), an analysis of the velocity of the COP can be expected to correspond with an analysis of jerk (third derivative of position). Therefore, one can expect to find a shorter latency for changes in the velocity of COP than for changes in the velocity of the foot. The idea that differences in response latency are largely the result of the signal that is used to determine the latency is in line with the evidence: the shortest latencies were found when relying directly on jerk (120 ms; Weerdesteyn *et al*. 2004) or on the velocity of COP (133 ms; our experiment), followed by a latency of 155 ms when relying on velocity (our foot data) and finally a latency of 200 ms when relying on position (Young & Hollands, 2012). For the latter study, a close look at their Fig. 2 reveals that the latency would have been about 170 ms if an extrapolation method rather than a threshold had been used. Therefore, the wide variety of behavioural measures of the latency of gait adjustments to changes in target position might mainly reflect differences between the methods of determining the latency rather than differences between the latencies themselves.

For the swing leg, we found a small response in the anterior direction for a lateral target shift, and in the posterior direction for a medial target shift (Fig. 5*A*). A similar small off‐axis response is visible in the stepping error reported by Barton *et al*. (2019; the upper‐right panel of Fig. 2). The initial COP response in the anterior‐posterior direction is also small, and in the same direction as the response of the swing leg. The substantial late (>200 ms) response of the COP in the anterior‐posterior direction was opposite to that of the initial response.

There was no significant difference between the left and the right leg for the amount of correction, but the latencies differed: the right leg responded 8 ± 15 ms earlier. All participants preferred to use the right leg to kick a ball, so the dominant leg appears to have an advantage over the non‐dominant leg in terms of responding fast to target shifts. In line with this result, a previous study observed that success rates were significantly higher for the dominant leg than for the non‐dominant leg for laterally shifted targets (Hoogkamer *et al*. 2015). To our knowledge, there have been no reports on asymmetries between limbs in response latencies, despite the many studies on handedness and sensorimotor control (reviewed by Goble & Brown, 2008).

To place the foot accurately onto a new position, not only the swing leg, but also the stance leg and the whole body need to adjust. In the related study of Barton *et al*. (2019), the whole‐body centre of mass (COM) moved diagonally (to the front and to the target‐shift direction). During the swing phase, the COM is moving from the stance leg to the swing leg side, and when it accelerates away from COP on the stance leg side, a moment of falling to the swing leg side is created (Reimann *et al*. 2017). In response to a lateral target shift, the whole‐body COM moved more to the lateral side of the swing leg. To compensate for this, the stance leg could create more forces in the opposite direction (towards the lateral side of the stance foot). This moved the COP in the opposite direction (discussed later as ‘ankle strategy’ to maintain stability).

In pilot experiments, we observed that the responses to large target shifts compromised stability in a visible way, which led us to use relatively small shifts. Compared to a target shift of 6.0 cm in the study of Barton *et al*. (2019), the smaller perturbation size (2.5 cm) in our study led to medio‐lateral adjustments that corrected a larger fraction of the perturbation. However, participants clearly did not fully correct for the medial target shifts. In our study, the mean correction to medial perturbations were significantly smaller than that to lateral perturbations (58% *vs*. 76%). This is in line with previous findings on a reach‐like stepping task (Reynolds & Day, 2005; Nonnekes *et al*. 2010) and is readily understandable as medial adjustments lead to a narrowing of the base of support during the double stance phase, compromising stability, while lateral positioning leads to a widening (Hof *et al*. 2005). Indeed, the response to medial perturbations is enhanced by adding support (Reynolds & Day, 2005; Nonnekes *et al*. 2010). In stroke patients the difference induced by absence of support was even more pronounced (large reduction in speed of medial movements), indicative of an active suppression of these responses.

For adjustments of arm movements, it has been found that responses to target shifts have 30–40 ms shorter latencies than responses to obstacles (Aivar *et al*. 2008, 2015). Therefore, we would have expected shorter latencies to changes in target position in our experiment than have been reported for responses to obstacle appearance (Weerdesteyn *et al*. 2007, 2004). Surprisingly, we found latencies to target shifts that are about 15 ms *longer* than the values that they reported, both for EMG and behaviour. Aivar *et al*. (2008, 2015) did not provide an explanation for their results, but suggested that it was their framing of the constraints that prioritised targets over obstacles. In a similar fashion, one might argue that the physical obstacles in the experiments of Weerdesteyn *et al*. (2007, 2004), which threatened balance, deserved a higher priority than the visual targets in our experiment. In contrast, in arm movements the obstacles do not represent a balance threat.

### Muscle responses

To our knowledge, our study is the first to show the changes in muscle activity in response to target shifts during walking. The fastest responses occur in muscles around the hip (GlM, latency about 123 ms). Responses occur slightly later, but still within 160 ms, in the other (more distal) muscles (Table 1 and Fig. 7). The early recruitment of GlM is consistent with the results of studies in which mechanical rather than visual perturbations were used (Hof & Duysens, 2013; Vlutters *et al*. 2018). We will discuss the various muscles in a proximal to distal order, and relate them to expectations based on muscle actions and the behavioural responses we observed.

The GlM contributes in the medio‐lateral direction because it acts as an abductor. Abduction of the swing leg moves the foot laterally, whereas abduction of the stance leg moves the COP laterally (MacKinnon & Winter, 1993). In response to a lateral target shift, both the foot (ipsilateral) and the COP (contralateral) responded in the lateral direction. For a perturbation in the medial direction, we found a suppression of the GlM of the contralateral (stance) leg. For this leg, the latency was about 123 ms for both types of response (Table 1 and Fig. 7). For the ipsilateral (swing) leg, one might have expected some suppression (less abduction yields adduction) but we only found a small facilitatory response. At any rate a suppression could not occur since there was no background activity in that period (see Fig. 6: up to 200 ms after the target shift there is no activity in the GlM of the swing leg that can be suppressed).

BF and ST are both biarticular muscles that extend the hip and flex the knee. In addition, ST elicits a small endorotation moment while BF is a foot exorotator. Exorotation causes a movement in the lateral direction of the middle of the foot of the swing leg (and in the medial direction for endorotation). In line with the function of the swing leg, we found that the ipsilateral ST was activated in response to a medial target shift and inhibited for a lateral target shift (Fig. 7). Surprisingly, i‐BF did not show the reverse pattern, but only responded with a small and late facilitation to medial perturbations.

Both vasti act as knee extensors, and thus have no direct role in medio‐lateral responses. However, as BF and ST flex the knee, we can expect both vasti to respond together with these muscles if knee stabilisation is required. This was indeed the case: they were activated almost synchronously with the ST and BF in the ipsilateral leg for medial target shifts.

The major function of both heads of the gastrocnemius is flexion of the knee and plantarflexion of the foot. In addition, GaM produces inversion while GaL induces eversion (Lee & Piazza, 2008). Reciprocal activation of these muscles thus produces inversion (or eversion). Such reciprocal activation of the two heads of the gastrocnemius has been observed following stimulation of cutaneous afferents at the ankle (Hauglustaine *et al*. 1998). Inversion of the stance foot contribute to a lateral adjustment of the COP (and eversion a medial adjustment). In line with the function for the stance leg, we found activation of the contralateral GaM in response to lateral target shifts, and suppression in response to medial shifts (Fig. 7), whereas GaL showed the opposite response. These reactions are functionally meaningful since lateral movement of the COP in the stance leg is appropriate for foot placement of the swing leg in the opposite direction. This ‘lateral ankle strategy’ is seen in many instances of medio‐lateral perturbations when the stance foot needs to resist an opposing force (Hof & Duysens, 2018; Reimann *et al*. 2018). In contrast, in the swing leg the GaL and GaM have no role for adjusting the foot, and consistent with this we found no responses in the heads of the ipsilateral gastrocnemius.

TA produces dorsiflexion and inversion. If the primary role was dorsiflexion, one would expect TA to be co‐activated with gastrocnemius to stabilise the ankle. If instead the inversion was the first objective, one would expect TA to be co‐activated with other foot invertors (such as GaM, see above). Inspection of Fig. 7 indicates that both occur for lateral perturbations. However, for the medial perturbations the responses in c‐TA are accompanied by suppression in c‐GaM and activation in c‐GaL, which argues against a role of c‐TA in inversion. In this case a role in stiffening the ankle is more likely.

An important question is whether the COP changes can be considered as anticipatory postural adjustments. If so then these COP changes should precede the major behavioural changes in the swing leg. This was clearly not the case. Instead, the COP responses almost coincided with the behavioural responses in the swing leg. The present results are more consistent with the triggering of a synchronous bilateral coordinated action. Such bilateral EMG activations are commonly seen in studies of gait perturbations (as summarised in Marigold & Misiaszek, 2009). Most examples described in that paper involved anterior‐posterior perturbations and responses in the lower leg. Our study shows that the basic principle of bilateral responses similarly applies for medio‐lateral perturbations and for the almost synchronous activation of hip muscles such as GlM. As to the functional significance of the stance GlM activation, Afschrift *et al*. (2018) explained that the stance GlM activation (following a platform translation) could tilt the pelvis upwards and thereby facilitate the motion of the swing leg. Alternatively, Hof & Duysens (2013) pointed towards a role in pelvis stabilisation.

### Neural mechanisms

In earlier studies the kinematic adjustments during the swing phase of gait initiation occurred about 120 ms after a medio‐lateral perturbation (Reynolds & Day, 2005). It was suggested that the fast responses might occur over a subcortical route. Later, the possible pathway was outlined. It involved a route over the cerebellum (reviewed by Potocanac & Duysens, 2017). This agrees well with cat reaching studies where evidence was found for collicular involvement (fast corrective movement were made with forelimb reaching adjustments induced by stimulation on superior colliculus; Courjon *et al*. 2004). In humans, evidence for a cerebellar contribution was found by Hoogkamer *et al*. (2017) who showed that fast adjustments during the swing phase were less accurately performed in patients with cerebellar lesions than in controls. The great benefit of the potential to make fast corrections was illustrated in the study by Potocanac *et al*. (2014 *a*), showing that such fast online corrections are still possible even after tripping.

An alternative is a fast route over the posterior parietal cortex (PPC) bypassing the motor cortex (see Fig. 4 in Potocanac & Duysens, 2017). In cats, PPC was found to contribute to online correction of visually guided locomotion, as the rapid neuron discharge in PPC could precede the fast corrections of limb trajectories, even in the absence of continual visual information (Marigold & Drew, 2011). However, later work in humans trying to disrupt online corrections by transcranial magnetic stimulation on effector‐specific regions of PPC failed to affect hand and foot corrections, suggesting those regions, in humans, might only affect the movement planning and not the corrections (Marigold *et al*. 2019).

In general, both pathways (subcortical and cortical) can possibly provide online corrections, meaning that during the swing phase there is a continuous comparison of current and desired position of the limb (as in reaching, see Smeets *et al*. 2016). In addition, later responses can be generated as well. These late responses are thought to be voluntary and to involve the typical motor cortex pathways (Potocanac & Duysens, 2017).

### Limitations

Our study was set up with fixed treadmill speed and target profiles, therefore the step length and width were unified, not individualised. These were defined based on average walking parameters for adults (Mazaheri *et al*. 2015), but might force some participants to adapt their baseline walking pattern in the familiarization period. Furthermore, our experiment was limited in terms of the number of muscles and body segments that we recorded. Therefore, we cannot provide a full description of the response strategies.

### Conclusion

During walking, human feet can adjust their trajectories online to changes in target position in a similar way to the arm. Those fast lower‐limb responses occur not only in the swing leg muscles, but also in the stance leg muscles.

## Additional information

### Competing interests

The authors claim no competing interests.

### Author contributions

The experiment was performed in the Movement and posture Analysis Laboratory Leuven, FaBeR, KU Leuven. Y.Z., J.S., S.V. and J.D. contributed to the design of the work; Y.Z. conducted and analysed data; all authors contributed to the data interpretation and editing of the work. All authors have approved the final version of the manuscript, and agree to be accountable for all aspects of the work in ensuring that questions related to the accuracy or integrity of any part of the work are appropriately investigated and resolved.

### Funding

The study is supported by DBOF scholarship, and the European Commission through MOVE‐AGE, an Erasmus Mundus Joint Doctorate programme (2011‐2015).

## Supporting information


**Statistical Summary Document**

**Movie S1**. Example video of task showing small shifts in stepping targets
