# Supplementary material for: Fast responses to stepping‐target displacements when walking
Source: J Physiol. 2020 Mar 27;598(10):1987–2000. doi: 10.1113/JP278986 (PMC7317495; doi:10.1113/JP278986)
Supplement: Supplementary file 1 — Statistical Summary Document [file TJP-598-1987-s001.docx]

**Manuscript Title:** Fast responses to stepping-target displacements when walking

Authors: Yajie Zhang, Jeroen B.J. Smeets, Eli Brenner, Sabine Verschueren, Jacques Duysens

**Animal model used, if applicable:** No.

**Underlying hypothesis:** This investigation tests the hypothesis that the leg muscle activation and foot kinematics during walking can be adjusted with a latency that is clearly less than 200 ms, and the hypothesis that medial and lateral adjustments are different.

**Definitions of ‘n’:**

n = number of participants.

**Statistical summary table:**

| Experimental question number* | Finding/ conclusion | Experimental location/ variable  e.g. cortex vs cerebellum or genotype | Mean value  (or other summary statistic) | SD | n (value) | P** | Units | Data comparisons  e.g. WT vs KO | Statistical test | Any other variable  e.g. subjects’ age or sex | Figure/table in which data are presented | Comments  e.g. observation |
| --- | --- | --- | --- | --- | --- | --- | --- | --- | --- | --- | --- | --- |
| 1. What is response latency (foot, COP, muscle activation)? | All latencies are clearly below 200 ms. | Foot Lateral-Left | 156 | 13 | 20 | - | ms | - | - | - | Table 1 | Observation. |
|  |  | Foot Lateral-Right | 152 | 10 | 20 |  | ms |  |  |  |  |  |
|  |  | Foot Medial-Left | 164 | 16 | 20 |  | ms |  |  |  |  |  |
|  |  | Foot Medial-Right | 153 | 13 | 20 |  | ms |  |  |  |  |  |
|  |  | COP Lateral-Left | 133 | 21 | 20 |  | ms |  |  |  |  |  |
|  |  | COP Lateral-Right | 127 | 24 | 20 |  | ms |  |  |  |  |  |
|  |  | COP Medial-Left | 145 | 22 | 18 |  | ms |  |  |  |  |  |
|  |  | COP Medial-Right | 129 | 27 | 20 |  | ms |  |  |  |  |  |
|  |  | i-GlM Lateral-Left | 126 | 9 | 20 |  | ms |  |  |  |  |  |
|  |  | i-GlM Lateral-Right | 120 | 8 | 20 |  | ms |  |  |  |  |  |
|  |  | c-GlM Lateral-Left | 123 | 11 | 20 |  | ms |  |  |  |  |  |
|  |  | c-GlM Lateral-Right | 121 | 12 | 20 |  | ms |  |  |  |  |  |
|  |  | c-GlM Medial-Left | 123 | 17 | 20 |  | ms |  |  |  |  |  |
|  |  | c-GlM Medial-Right | 123 | 25 | 20 |  | ms |  |  |  |  |  |
|  |  | i-ST Lateral-Left | 143 | 22 | 20 |  | ms |  |  |  |  |  |
|  |  | i-ST Lateral-Right | 136 | 22 | 20 |  | ms |  |  |  |  |  |
|  |  | i-ST Medial-Left | 137 | 19 | 20 |  | ms |  |  |  |  |  |
|  |  | i-ST Medial-Right | 130 | 23 | 20 |  | ms |  |  |  |  |  |
| 2. Does the correction differ between directions and/or between legs? | Medial correction is less than lateral correction. No difference between left and right leg. | Correction Lateral-Left | 77 | 18 | 20 | ML: **p<0.001**  LR: p=0.931 | % | Medial vs. Lateral  Left vs. Right | 2-way ANOVA |  | text |  |
|  |  | Correction Lateral-Right | 75 | 20 | 20 |  | % |  |  |  |  |  |
|  |  | Correction Medial-Left | 57 | 14 | 20 |  | % |  |  |  |  |  |
|  |  | Correction Medial-Right | 59 | 13 | 20 |  | % |  |  |  |  |  |

*You may use multiple lines for the same question to indicate multiple comparisons

** Authors may wish to make the text bold where p is considered significant against a stated confidence limit
